# The Week in the Courts

**Published:** 1911-04-08

**Authors:** 


### The Week in the Courts.

### AN UNCERTIFIED MIDWIFE.

At Windsor Police Court Amy Anietta Pope was summoned for infringement of the Midwives Act, in that she attended women in childbirth otherwise than under the direction of a qualified medical practitioner, she not being certified by the Midwives Board. Defendant pleaded guilty, and on promising not to offend again, was dealt with under the Probation of Offenders Act.

In the High Court of Justice, Chancery Division, before Mr. Justice Parker, Mr. Romer, K.C., with Mr. Eastwood, appeared on behalf of The London and Counties Medical Protection Society, Limited, in support of a petition asking the Court to sanction a proposed alteration in the Memorandum of the Association of the Company. Mr. Romer explained that the original memorandum seemed to restrict rather unduly the general purposes of the Society, and it was desired to have that absolutely set at rest by this new memorandum, which really gives power to the Society only to do those kinds of things which societies of this description are accustomed to do, and to do them with greater efficiency. Mr. Romer explained that the financial position of the Society was highly satisfactory, the available assets of the Society amounting to about £12,000, and the debts of the Society being practically nothing. The resolutions altering the memorandum had been carried in accordance with the Act at two meetings of which due notice had been sent to all the members of the Society. His Lordship made the order asked for. Under the new Memorandum and Articles of Association of The London and Counties Medical Protection Society, which have now come into force, a member's subscription, when it falls due, will henceforward be £1 instead of 10s. as heretofore, and from the date on which it reaches the higher rate of £1 he will be entitled to the increased benefits. If he wishes to be entitled to the increased benefits under the amended Memorandum and Articles of Association from February 14, 1911, to the date on which his subscription is renewable, he may pay now a proportion of the additional subscription (10s.) from February 14. 1911, to the date of renewal, namely, the anniversary of his election as a member of this Society. The privileges of members remain exactly as before with respect to the payment by the Society of the whole of the costs of conducting for members any cases which the Society undertakes for them; but, with the increased subscription, the Society will also pay costs of the other side and damages, up to an amount together not exceeding £2,000 for any one member in any one year, when costs or damages are awarded against members in cases in which the Society has been unsuccessful. The Society will itself bear the aggregate loss, arising from payment on behalf of the members of damages and costs of the other side, up to £2,000 in any one year, and it will insure against any loss of the kind, over and above £2,000, up to the amount of an additional £20,000 in any one year. The payment of costs of the other side and damages will not apply in cases taken up by the Society at any time before the increased subscription, or the proportion thereof before referred to, has been paid by members.

